# Changes in landscape and climate in Mexico and Texas reveal small effects on migratory habitat of monarch butterflies (*Danaus plexippus*)

**DOI:** 10.1038/s41598-024-56693-z

**Published:** 2024-03-20

**Authors:** Jay E. Diffendorfer, Francisco Botello, Mark A. Drummond, Zach H. Ancona, Lucila M. Corro, Wayne E. Thogmartin, Peter C. Ibsen, Rafael Moreno-Sanchez, Laura Lukens, Victor Sánchez-Cordero

**Affiliations:** 1 U.S. Geological Survey, Geosciences and Environmental Change Science Center, Lakewood, CO USA; 2 Departamento de Zoología, Instituto de Biología, Universidad Nacional Autónoma de México, Mexico City, Mexico; 3 Departamento de Monitoreo Biológico y Planeación de Conservación, Conservación Biológica y Desarrollo Social, Mexico City, Mexico; 4 U.S. Geological Survey, Upper Midwest Environmental Sciences Center, La Crosse, WI USA; 5 Department of Geography and Environmental Sciences, University of Colorado Denver, 1200 Larimer St, NC 3016-C, Denver, CO 80204 USA; 6 Monarch Joint Venture, 2233 University Ave W., Suite 426, St. Paul, MN USA; 7 Department of Forestry & Rangeland Science, Colorado State University, 1472 Campus Delivery, Fort Collins, CO USA

**Keywords:** Climate change, Ecology, Animal migration, Conservation biology, Population dynamics, Ecology, Animal migration, Climate-change ecology, Conservation biology, Population dynamics, Environmental sciences, Environmental impact

## Abstract

The decline of the iconic monarch butterfly (*Danaus plexippus*) in North America has motivated research on the impacts of land use and land cover (LULC) change and climate variability on monarch habitat and population dynamics. We investigated spring and fall trends in LULC, milkweed and nectar resources over a 20-year period, and ~ 30 years of climate variables in Mexico and Texas, U.S. This region supports spring breeding, and spring and fall migration during the annual life cycle of the monarch. We estimated a − 2.9% decline in milkweed in Texas, but little to no change in Mexico. Fall and spring nectar resources declined < 1% in both study extents. Vegetation greenness increased in the fall and spring in Mexico while the other climate variables did not change in both Mexico and Texas. Monarch habitat in Mexico and Texas appears relatively more intact than in the midwestern, agricultural landscapes of the U.S. Given the relatively modest observed changes in nectar and milkweed, the relatively stable climate conditions, and increased vegetation greenness in Mexico, it seems unlikely that habitat loss (quantity or quality) in Mexico and Texas has caused large declines in population size or survival during migration.

## Introduction

The monarch butterfly (*Danaus plexippus*), an organism weighing only one-half gram, is venerated for an annual migration in North America that may span 5000 km. The eastern migratory population passes through Texas and northern Mexico twice during its annual cycle. Once as individuals migrate from Canada and the US to overwintering areas in montane fir forests in central Mexico and then again as adults who survived the winter fly northward each spring, laying eggs primarily in Texas before they die. From here subsequent generations migrate northward spreading across central and eastern US, and southern Canada to begin the cycle anew. Declines over the last 3 decades in this migratory population created large public concern, prompting considerable research and conservation actions^1,2^.

**Figure 1:**
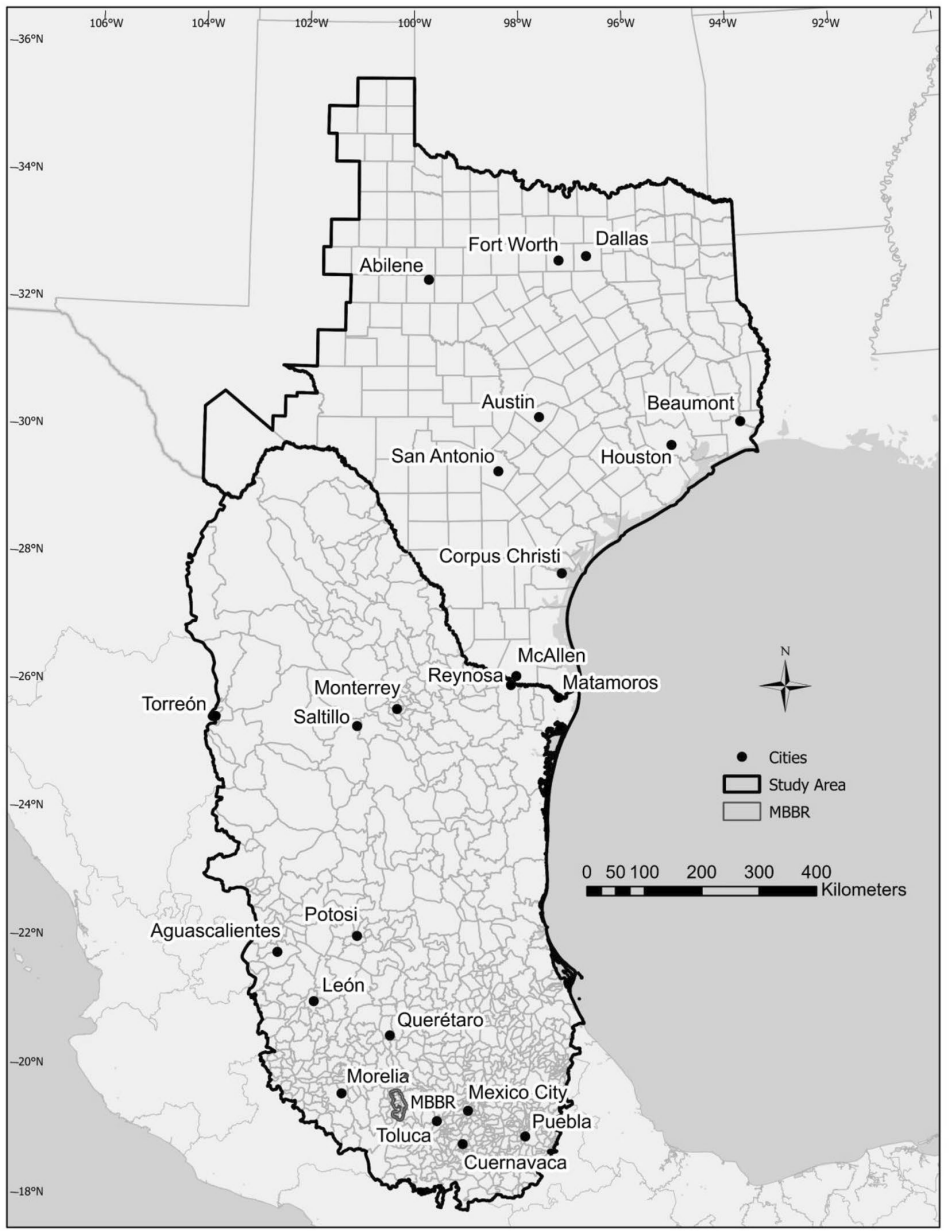
Study extent in Mexico (with municipalities) and Texas (with counties) created using ArcGIS Pro 3.2.1, Copyright © 1995–2023 Esri (https://www.esri.com/en-us/arcgis/products/arcgis-pro/overview). MBBR is the outline of the Monarch Butterfly Biosphere Reserve and represents the general region where monarchs overwinter.

Areas traversed during migration in Mexico and Texas (Fig. 1) support monarchs in a variety of ways^3,4^. During fall migration, monarchs utilize nectar resources in this region to sustain their migration and build fat reserves necessary to survive the winter in central Mexico^5,6^. In the spring, monarchs utilize nectar sources to migrate north through Mexico and for mating and laying eggs on milkweed in Texas^7^, and possibly some regions of northern Mexico^4,8^.

The annual life cycle, population dynamics, and the causes of the eastern monarch population decline are not fully understood. Annual variation in overwintering abundance (measured as the area occupied by overwintering clusters of monarchs^9^), is driven by linked spatio-temporal generations^10–12^. For example, climate conditions at various stages of the life cycle influence seasonal, annual, and possibly longer-term, trends^13–16^. Weather effects on spring recruitment (primarily in Texas) can influence overall annual population dynamics^13,17^ while drought during the fall migration affects monarch lipid levels crucial for overwintering survival^6^, subsequent spring breeding^18,19^, and may influence overwintering population size^20^.

The eastern population declined from the 1990s to 2014, with no statistically detectable trend from 2014 to 2018^21^ and recent analyses indicate the population has been relatively stationary, but low, for the last 10 years^22^. Population declines in the mid-1990s and early 2000s were associated with the rapid adoption of herbicidally tolerant GMO corn and soybeans across the midwestern US from ~ 1994 to ~ 2006 and glyphosate application, which caused large declines in milkweed and monarch butterfly egg production^10,23,24^. These findings support the “milkweed limitation hypothesis”, (MLH), which posits the decline in milkweed has lowered the carrying capacity of the monarchs’ breeding habitat^25^, driving both the observed population decline and the more recent stationarity around a lower population size after milkweed was essentially eliminated from agricultural fields in the midwestern US^16,21^.

Other processes, beyond a decline in breeding habitat, affect monarch populations and could also contribute to historical declines and/or affect current population dynamics. The “migration survival hypothesis” (MSH) suggests survival during the fall southward migration may be declining, because breeding populations estimated from summer surveys in the US have remained stable, yet overwintering numbers have declined^12,26^. Taylor et al.^27^, based on monarch tagging data, argued migration success has not declined, but concerns exist about this conclusion^28^. Others have argued the lack of observed summer trends in the breeding region stems primarily from a sampling design that does not account for loss of monarchs in agricultural lands^24,29–31^.

A variety of mechanisms could cause declines in fall migration success^1^. Compelling evidence indicates parasitism by *Ophryocystis elektroscirrha* (OE) reduces migration survival^32,33^ and that OE infection rates have increased since the mid-2000s^34^. Two studies document monarch vehicular collision mortality in Texas^35^ and Mexico^36^. Migratory monarchs may stop their migration, break reproductive diapause, and begin laying eggs when they encounter tropical milkweed (*Asclepias curassavica*), which does not seasonally die back in coastal Texas, but the extent of tropical milkweed impact to the population is not yet known^37^. Nectar sustains migration, and changes in the quantity, quality, or timing of nectar could affect migration success. Saunders et al.^20^ but not Zylstra et al.^16^ positively correlated fall vegetation greenness (the Normalized Difference Vegetation Index, NDVI) in the US with winter monarch abundance. Changing climate and land use could affect migration success via multiple pathways and recent studies indicate longer term changes in climate may contribute to the long-term declines in the eastern monarch population^13,16,20^.

Land-use land-cover (LULC) studies describe how land cover, and land use by humans, changes through time and the causes/consequences of those changes. LULC analyses have tracked the status of oyamel fir (*Abies religiosa*) forests supporting monarchs in Mexico^38–42^, identified restoration potential of monarch habitat in the US^43–45^, modelled monarch and milkweed distributions^7,46,47^, migratory connectivity^48^ and threats during migration^49^. Analyses of how LULC may impact monarch habitat in Texas and Mexico, have not, to date, occurred, despite the important role this region plays in the eastern population’s life cycle.

This critical omission in understanding suggests we should investigate LULC in Mexico and Texas. Deforestation for cattle grazing, and cropland expansion in irrigated desert regions occurred in Mexico from 1993 to 2014^50,51^. In Texas, milkweed on rangelands appears widespread^52^ but urbanization may encroach on rangelands in south Texas by 2050^53^. The increase in urban land area may impact connectivity of natural habitats through road construction fragmenting monarch habitat and resulting in heightened mortality from vehicle collisions^35^.

We analyzed spatio-temporal trends in LULC, nectar and milkweed resources, and climate variables in northern Mexico and Texas and related the results to hypotheses about milkweed limitation during the first spring generation, long-term trends in climate that could affect population dynamics, and changes in habitat that could alter fall migration survival. We combined LULC analyses and Monte Carlo methods to estimate changes in the quantity of spring milkweed and fall/spring nectar resources. To assess change in habitat quality in the fall and spring, we estimated changes in climate variables associated with plant growth and productivity, as well as the NDVI, a remotely sensed index of vegetation greenness.

## Results

### Land cover trends

**Figure 2:**
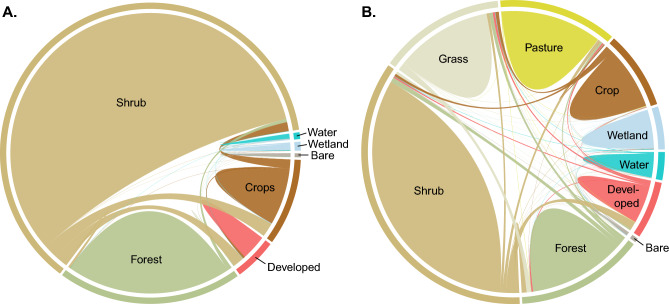
Chord diagram showing land cover changes across the study area in Mexico (**A**) from 2000 to 2020 based on Global Land Cover and Discovery (GLAD) data and Texas (**B**) from 2001 to 2019 based on National Land Cover Database (NLCD). For Texas, individual Forest, Developed, and Wetland classes were combined into single categories to enhance legibility. The same color leaving a cover class to another represents a transition from that class to the other. GLAD classes were labeled akin to NLCD for ease of interpretation across figures. A crosswalk between GLAD and NLCD is available in Supplementary Data 1.

From 2001 to 2020, shrublands dominated landcover in both Texas and Mexico (Table 1, Fig. 2) while crops and developed lands increased through time (Table 1). Notably, developed lands, or most of the developed subclasses, increased >~ 50% (Table 1).

**Table 1:** Land cover change in Mexico and Texas (km^2^). Net change is Gain minus Loss whereas Gross is Gain plus Loss.

Country/State and land cover class	Start year	End year	Loss	Gain	Net	Gross	Annual	Percent annual change	Total percent change
Mexico	2000	2020							
Bare	2759	2650	141	32	− 109	173	− 6	− 0.21	− 3.9
Shrub/Herbaceous	393,630	382,501	20,507	9378	− 11,129	29,885	− 586	− 0.15	− 2.8
Tree cover	126,023	124,592	3404	1973	− 1431	5377	− 75	− 0.06	− 1.1
Wetland herb	4970	4381	1178	589	− 590	1767	− 31	− 0.62	− 11.9
Wetland tree	599	626	66	93	27	158	1	0.24	4.5
Water	3538	4053	630	1146	515	1776	27	0.77	14.6
Cropland	49,496	51,423	8914	10,840	1927	19,754	101	0.20	3.9
Built-up	18,869	29,659	0	10,790	10,790	10,790	568	3.01	57.2
Texas	2001	2019							
Bare	2251	1922	690	361	− 329	1051	− 18	− 0.81	− 14.6
Cropland	43,589	47,101	1290	4802	3512	6092	195	0.45	8.16
Dev. High	2306	3396	0.4	1090	1089	1090	61	2.62	47.2
Dev. Low	9285	10,421	495	1631	1136	2125	63	0.68	12.2
Dev. Med	5378	8127	35	2784	2749	2819	153	2.84	51.1
Dev. Open	13,553	13,652	1255	1355	100	2609	6	0.04	0.7
Deciduous	18,983	18,105	20,590	1181	− 878	3239	− 49	− 0.26	− 4.6
Evergreen	37,933	38,246	4847	5160	313	10,007	17	0.05	0.8
Mixed	14,837	14,401	1733	1297	− 435	3030	− 24	− 0.16	− 2.9
Grass	72,387	69,922	8196	5731	− 2465	13,927	− 137	− 0.19	− 3.4
Pasture	72,955	67,612	5875	532	− 5343	6407	− 297	− 0.41	− 7.3
Shrubland	193,272	192,920	8012	7659	− 352	15,671	− 20	− 0.01	− 0.2
Water	16,003	16,969	394	1359	965	1752	54	0.34	6.0
Wetland herb	7066	6995	1288	1217	− 71	2506	− 4	− 0.06	− 1.0
Wetland woody	18,875	18,883	921	930	9	1850	0	0.00	0.1

Annual is annualized net change. Land cover class labels follow the datasets used for Mexico and Texas. Herbaceous is shortened to herb; *Dev.* developed.

In Mexico, the growth of developed areas and croplands caused a − 2.8% decline in shrubland (Fig. 2a). Forest to shrubland, croplands to shrubland, and barren to shrubland transitions also occurred but at lower rates than LULC away from shrubland. Transition to water as lakes filled and the loss of wetlands to croplands caused a decline in wetland shrub of − 11.9%.

In Texas, shrublands were primarily changed to developed, pasture, and grasslands, though the overall percent change was low (Table 1). Deciduous forests (− 4.6%), and pastures/hay (− 7.3%) also declined. For shrublands and grasslands the areal coverage in 2019 was slightly less than in 2001 (− 0.18% for shrublands, and − 3.4% for grasslands, Table 1 and Fig. 2b).

To assure land cover data used in Mexico (Global Land Cover and Discovery (GLAD)) and Texas (National Land Cover Database (NLCD)), measured LULC similarly, we compared statewide trends in gross land cover change across Texas, where both datasets were available from 2000 and 2020 for GLAD and 2001 and 2019 for NLCD. Overall trends were similar (Supplementary results, Fig. S1). County-level differences in levels of gross change could be attributed to differences in the classification systems between GLAD and NLCD. GLAD combines all the NLCD forest classes into a single class, and the GLAD shrubland class included grasslands, pastures, low-intensity and urban open space, barren lands and low-percent-cover forests based on a crosswalk of GLAD and NLCD in Texas (Supplementary Data 1).

### Milkweed and nectar resources

Data describing nectar and milkweed resources differed between Mexico and Texas, requiring elicitation of expert opinion for nectar and milkweed resources in Mexico and synthesis of published and unpublished literature in Texas. Across both Mexico and Texas, information on nectar resources was somewhat similar, varying from comparatively low value (~ 0.2) to moderately valuable (~ 0.5) among land cover classes (Table 2). Differences between fall and spring in the value of individual land covers to monarchs were greater in the more temperate US compared to Mexico. Mexico experts considered agriculture, scrublands, and urban as supporting the highest spring nectar resources, with forests and shrubs having the highest values in the fall. For Texas, shrublands, grasslands, and herbaceous wetlands had the highest spring nectar values, while deciduous forests and shrublands were scored highest in the fall. Experts in Mexico gave similar milkweed values (0.23–0.29) across all land cover types except the lower-valued wetland herbaceous land cover. Across Texas, field-collected milkweed density varied an order of magnitude (~ 6 to 60) across land cover classes. Grasslands, deciduous forests, and developed open space had the highest milkweed densities, while evergreen forests, and dense urban development had the lowest (Table 2).

**Table 2:** Nectar and milkweed resource values by land cover classes for Mexico and Texas, including means (and standard deviations) from the modelled distributions. Values for Mexico were estimated from surveys. Water was zero for both countries.

Land cover class	Fall nectar	Spring nectar	Milkweed
Mexico			
Shrub/Herbaceous	0.414 (0.235)	0.447 (0.272)	0.233 (0.170)
Cropland	0.439 (0.254)	0.418 (0.293)	0.263 (0.212)
Wetland herb	0.201 (0.119)	0.237 (0.134)	0.146 (0.120)
Wetland tree	0.380 (0.222)	0.411 (0.251)	0.267 (0.204)
Bare	0.332 (0.215)	0.402 (0.205)	0.237 (0.180)
Built-up	0.410 (0.253)	0.418 (0.274)	0.251 (0.199)
Tree cover	0.404 (0.249)	0.474 (0.272)	0.287 (0.226)
Texas			
Developed open space	0.460 (0.254)	0.461 (0.293)	28.5 (58.0)
Developed low intensity	0.477 (0.242)	0.533 (0.185)	9.6 (23.0)
Developed med intensity	0.393 (0.240)	0.402 (0.235)	6.2 (17.9)
Developed high intensity	0.263 (0.178)	0.317 (0.210)	3.3 (8.5)
Barren	0.22 (0.124)	0.25 (0.133)	0
Deciduous forest	0.399 (0.328)	0.714 (0.281)	37.4 (185.1)
Evergreen forest	0.366 (0.254)	0.438 (0.299)	6.2 (18.0)
Mixed forest	0.378 (0.324)	0.594 (0.325)	50.8 (142.7)
Shrub	0.456 (0.325)	0.685 (0.234)	8.9 (46.2)
Herbaceous grassland	0.491 (0.383)	0.574 (0.334)	66.8 (195.5)
Pasture/Hay	0.366 (0.223)	0.298 (0.252)	21.6 (118.3)
Crops	0.329 (0.286)	0.308 (0.286)	0
Woody wetlands	0.434 (0.299)	0.587 (0.193)	0
Herbaceous wetlands	0.458 (0.340)	0.463 (0.201)	0

Values for Fall and Spring Nectar in Texas were from Koh et al. 2016. Milkweed values in Texas were estimated from samples as plants/ha. Land Cover Class labels follow the datasets used for Mexico and Texas.

### Quantitative change in milkweed and nectar resources

Combining LULC with estimates of nectar and milkweed values, resulted in an estimate of a − 2.9% decline in milkweed density in Texas between 2001 and 2019 (starting from ~ 1.04 billion plants in 2001), and no change, or a slight increase, in the milkweed index in Mexico from 2000 to 2020 (Table 3). Nectar resources declined < 0.2% in the fall and spring in both Mexico and Texas.

**Table 3:** Net change in milkweed and nectar resources from 2000–2020 in Mexico and 2001–2019 in Texas.

Country/State	Change	Fall nectar	Spring nectar	Milkweed
Mexico	Value	− 8.6 (47.9)	− 333.4 (50.1)	267.9 (35.0)
	Total percent	− 0.003 (0.019)	− 0.12 (0.018)	0.16 (0.021)
Texas	Value	− 304.3 (28.8)	− 313.3 (29.1)	− 29,981,000 (1,183,480)
	Total percent	− 0.14 (0.01)	− 0.11 (0.01)	− 2.88 (0.11)

Data are presented as means (SE in parenthesis) of both raw values and total percent change relative to the first year of the time interval. Note that total percent change is calculated from the start and end years of the interval for each country/state and is not an annual rate of change. For Texas, milkweed is reported as total plants across the study extent. All other values were derived from expert opinion and represent resource value change per km^2^.

### Geographic patterns of milkweed and nectar change

**Figure 3:**
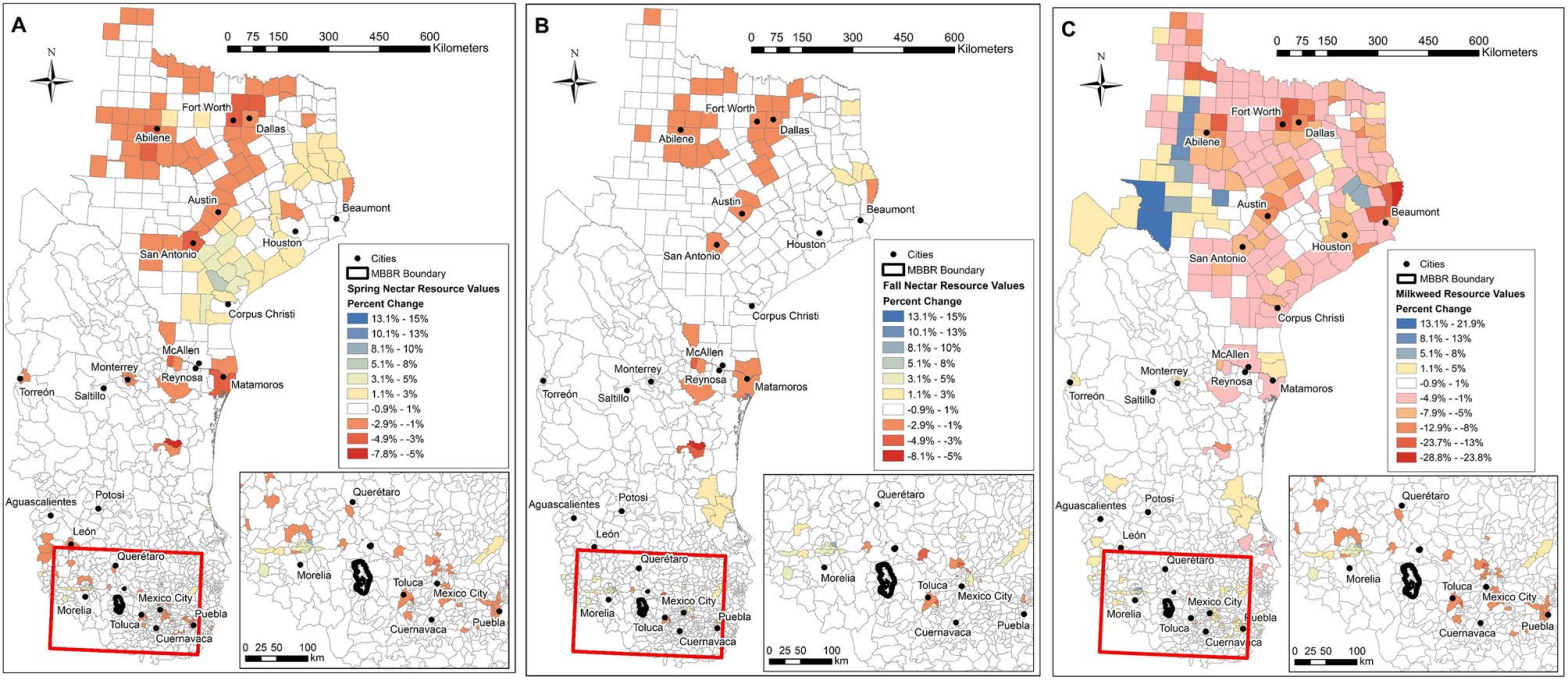
Patterns of percent change across the study extent for spring nectar (**A**), fall nectar (**B**), and Spring milkweed (**C**). Resource change was summed at the county level in Texas and the municipality level in Mexico created using ArcGIS Pro 3.2.1, Copyright © 1995–2023 Esri (https://www.esri.com/en-us/arcgis/products/arcgis-pro/overview).

Most counties (Texas) and municipalities (Mexico) experienced small changes in nectar and milkweed resources (Fig. 3a–c) but there were cases of positive and negative change. Spatial patterns in habitat change indicated more change in Texas than Mexico, and more change (primarily loss) in milkweed than nectar.

In Texas, counties along the I-35 highway corridor connecting San Antonio, Austin, and Dallas-Fort Worth, have growing urban areas, and showed declines in spring and fall nectar of 1–5% and milkweed of 4–25%. Counties in central Texas near Abilene exhibited declines in nectar of 1–3% primarily in the spring. One to 5% gains in spring nectar occurred in counties in eastern Texas, while very few counties gained fall nectar. Milkweed density declined 1–5% across most of the Texas study extent, except in the western (mainly southwestern) counties, where relatively large gains of 1–21% occurred, possibly caused by large wildfires converting shrublands to grasslands. Counties showing declines in milkweed density > 15% were along the I-35 highway corridor, the far eastern border near Beaumont, and in a few counties in the north-western parts of the study extent.

In Mexico, eastern municipalities along the US-Mexico border near Reynosa and Matamoros and a small number of additional municipalities demonstrated declines in spring and fall nectar resources. Municipalities around the overwintering locations had both increases and decreases in spring and fall nectar resources. Milkweed resource value increased 1–5% in a few southern municipalities around the overwintering location, while more northern municipalities, especially those near the US-Mexico border had declines between − 1 and − 7.9%.

### Qualitative change in resources based on climate variables and NDVI

In Mexico both fall and spring NDVI increased from 1997 to 2021 (Fall, β_year_ = 0.0026, SE_year_ = 0.0007, t = 3.38, p = 0.003; Spring, β_year_ = 0.0026, SE_year_ = 0.0007, t = 3.80, p = 0.0009, Supplementary results, Fig. S2). The other environmental variables did not demonstrate statistically significant (p < 0.05) trends through time, though spring average minimum temperatures increased with marginal statistical significance (β_year_ = 0.310, SE_year_ = 0.158, t = 1.97, p = 0.059, Supplementary results, Fig. S2).

Across the Texas study extent, none of the environmental variables showed statistically significant trends from 1992 to 2021 at p < 0.05 (Supplementary results, Fig. S3). Marginal changes occurred in several variables; for instance, Fall NDVI increased (β_year_ = 0.002, SE_year_ = 0.0008, t = 1.72, p = 0.10), spring Palmer Drought Severity Index (PDSI) decreased (β_year_ =  − 8.30, SE_year_ = 4.75, t =  − 1.75, p = 0.092), and spring maximum temperature increased (β_year_ = 0.54, SE_year_ = 0.302, t = 1.79, p = 0.084).

## Discussion

Changing land cover and climate are fundamental processes driving the environmental conditions affecting species distributions and their population dynamics^54,55^. We measured only small changes in land cover, which resulted in small to no expected changes in nectar and milkweed resources across Mexico and Texas. We also observed few substantive temporal trends in annual climate, aside from an increasing trend in NDVI in Mexico in fall and spring.

### Land cover change and monarch habitat

During ~ 2000–2020, the patterns of LULC across the study extent were typical of those observed in other parts of North America where anthropogenic land cover increased, while natural land cover types declined^56^. In Mexico, shrublands declined by ~ 3% while in Texas, none of the natural vegetation classes declined more than 5%, though forests in eastern Texas, where rotational harvest occurs^57^, had the largest declines.

The estimated change in milkweed and nectar resources were consistent with the relatively low levels of loss in natural land cover classes and patterns of LULC dynamics affecting vegetation. Our results indicate small, to no, net changes in nectar and milkweed in Texas and Mexico from 1992 to 2021. Milkweed in Texas declined ~ 2.9%, and both milkweed and nectar resources, in fall and spring, showed spatial heterogeneity in trends through time.

In the midwestern US, trends in milkweed were estimated using a combination of field surveys and geographic extrapolations of adoption rates of glyphosate-tolerant corn and soybeans^23,58,59^. Pleasants et al.^58^ calculated a loss of 850 million milkweed stems in corn and soybeans, and an additional 11 million lost from changes to land cover in the Midwest, while Flockhart et al.^10^ estimated 1.49 billion stems were lost across the entire eastern US monarch breeding range. In non-agricultural lands of the US Midwest, milkweed abundance has likely been stable^59^.

Unlike the US Midwest, we did not detect a change in milkweed in Mexico and estimated a − 2.9% decline in Texas from 2001 to 2019, representing an estimated ~ 29 million plants lost relative to ~ 1.04 billion existing in 2001 across ~ 53 million ha. Our milkweed estimates in Texas are low compared to the 233 million to 1.3 billion plants estimated on just 2.8 million ha of rangelands in Texas^52^. Many of the LC classes in Texas support far fewer milkweeds than rangelands, so we expect our estimates, on a per area basis, to be smaller than Spaeth et al.^52^. Flockhart et al.^10^ estimated ~ 1.7 billion stems across the entire southern US, including Texas, indicating our estimate falls between Flockhart et al.^10^ and Spaeth et al.^52^.

The impacts of LULC on the eastern migratory population depends on how monarch densities vary across space relative to patterns of increasing or decreasing habitat. Our work indicates there may be geographic concordance between areas in Texas most suitable for monarchs and higher rates of habitat loss. For example, central Texas counties dominated by urbanization and eastern Texas showed larger declines in nectar and milkweed. These same regions may have the highest spring suitability for adults^3^ and the urban counties around San Antonio-Austin and Dallas-Fort Worth were most utilized by caterpillars^7^. More formal analysis of these patterns is needed, but this concordance suggests strategies to enhance habitat in urban areas^44,60^ and rights of ways in central Texas may help counter the conversion of rangelands and shrublands to urban in these areas.

Mexico showed less overall change in monarch habitat and less spatial heterogeneity in change than Texas, particularly for milkweed. Annual rates of LULC were generally similar across land cover classes for both Mexico and Texas (Table 1). However, in Mexico, experts estimated smaller differences in nectar and milkweed resource values across land cover types than in the US so the largest land cover transitions (shrub to urban, shrub to agriculture) represented relatively smaller changes in monarch resource values in Mexico than in the US. Finally, GLAD in Mexico combines the NLCD grass and shrub classes into a single land cover category and could not estimate changes in habitat caused by transitions between these two cover types; thus, transitions between these cover classes and the difference in value they represent to monarchs could not be captured.

### Temporal changes in climate and NDVI

Climate variables showed high levels of annual variability, but the only substantial temporal trend we observed was increased NDVI in both fall and spring in Mexico. The lack of statistically significant trends in Texas runs counter to statewide warming and increased precipitation in eastern Texas observed in longer time series^61^. Our results indicate that during the ~ 30-year time span matching declines in monarch abundance, the climate variables we measured show few to no changes relative to high levels of annual variability. Saunders et al.^20^ found a positive relationship between overwintering colony size and fall NDVI in a region covering northeast Texas, Oklahoma, Kansas, Arkansas, Louisiana, and Mississippi. Zylstra et al.^16^, however, focusing on a larger region of the US (90° W to 105° W, 30° N to 40° N) found no relationship between NDVI and overwintering colony size. Both studies used MODIS data, which became available in 2000. We used 30-m LANDSAT derived NDVI from Google Earth Engine (GEE), which is available annually from 1984. Future modelling approaches that include climate variables from Mexico during fall and spring migration would be helpful to determine if they influence population dynamics.

How do our results relate to hypotheses and mechanisms of monarch decline and population dynamics? First, milkweed limitation in Texas does not appear to be a driver of population dynamics, likely because Texas is not heavily dominated by agriculture. Our results, in addition to Spaeth et al.’s^52^ estimates, indicate milkweed abundance in Texas has declined, but not to the extent observed in the US Midwest, and that milkweed limitation is unlikely for the first generation of breeding monarchs.

Second, our results indicate loss of migratory habitat in Texas and Mexico may not be a mechanism for potential declines in migration success. We saw little to no signal in the nectar resources that would indicate a decline in fall migration survival and an unexpected increase in NDVI during the fall and spring in Mexico, suggesting the possibility that nectar resources have increased over the last 23 years rather than diminished.

Phenological shifts in milkweed and floral availability may have generated a mismatch between the timing of spring and fall migration^62^ and resource availability for monarchs. A separate study is needed to understand if milkweed and nectar phenology has shifted away from the timing of the monarch migration through Mexico and Texas. However, our results indicate that milkweed and floral availability has been predictable for fall and spring migrating monarchs through Mexico and Texas over the last 20–30 years. Migrating monarchs may opt to become residents and take advantage of tropical milkweeds in this region^37^. The monitoring of resident monarch populations in this region will help to test this possibility.

One implication of the migration survival hypothesis is that declines in fall migration survival, large enough to cause a decline in the wintering population, require a commensurate increase in reproductive output in the spring to generate a stable summer breeding population. Furthermore, as the wintering population declined, the difference between the summer breeding population and the winter population increased, so the magnitude of reproductive output necessary to overcome declining fall survival must also have increased year-over-year.

Perhaps the positive trend in spring NDVI in Mexico supports enhanced female spring migration survival from overwintering areas to first-stage breeding grounds and allows greater egg production by females. We are skeptical of this possibility because our data do not show that floral availability has declined in fall and increased in spring, but instead likely increased in both seasonal environments. Ultimately, it is difficult to reconcile how the trends (or the lack thereof) in land and climate change we observed would simultaneously lead to decreased fall migration success yet support or enhance spring migration and reproductive output in equal but increasing measure each year.

### Study limitations

The datasets we used can measure shifts in the dominant LULC classes, but the data used cannot adequately map more subtle changes in landscapes like crop margins^48^, nor changes within each land cover class. For example, degraded shrubland sites had different temporal patterns of NDVI than intact sites^63^. Given these issues the LULC, nectar, and milkweed resources analyses were designed to identify large changes in habitat and map them at coarse spatial resolutions^64^. Given the categorical nature of the LULC analyses, these efforts focused on changes in habitat quantity, and we used the climate variables and NDVI, to assess possible changes in habitat quality.

Given the conservation efforts related to butterflies, pollinators, and arthropods in general, developing empirical relationships between host plants, nectar resources, and remotely sensed data would allow broad-scale and historical analyses of these resources and link them to species of interest/management concern. Our Monte Carlo based approach provides a robust framework for integrating LULC data and field collected survey information. Previous studies indicate field campaigns over large regions are feasible^52,59,65^ and analyses of the Integrated Monarch Monitoring Program^66^ data will hopefully generate milkweed densities for LULC classes over a much broader region than our Texas analysis.

Our results indicate monarch habitat in Mexico and Texas is more intact than the agricultural landscapes of the midwestern US. However, milkweed in Texas has declined, and may continue to do so if existing LULC dynamics persist. As such, efforts to conserve and restore milkweed in Texas, especially in central Texas, where both urbanization and higher densities of monarchs may overlap, may offset some losses.

To the best of our knowledge, this research is the first monarch study to analyze trends in climate and NDVI across migratory habitat in Mexico and the observed increasing trends in NDVI were unexpected. Incorporating this information into models of eastern monarch population dynamics would help us better understand if and how spring and fall conditions in Mexico affect population dynamics. A next step is to understand how much habitat is currently protected from future land cover changes, resilient to climate change, and if these protected areas would be sufficient to support monarch migration and reproduction and generate similar benefits to other species. We anticipate continuing changes to climate, so understanding climate-related changes in monarch, floral, and milkweed distributions will inform decisions regarding how and where to best conserve existing migratory habitat.

## Materials and methods

Our methodological choices were based, in part, on data availability. For example, milkweed and nectar densities are not well-sampled across our study extent, and land cover data vary between the US and Mexico^64^. We used land cover datasets from Mexico and Texas to estimate land cover change, then used Monte-Carlo methods to link changes in land cover to changes in monarch habitat (spring and fall nectar and milkweed resources) using empirical data and surveys of experts. In addition, we assessed temporal trends in climate variables and NDVI across the study extent in Mexico and Texas to determine if climate conditions that affect plant growth and nectar production, as well as an index of vegetation condition, changed through time. NDVI measured vegetation greenness and recent studies suggest it, or the related Enhanced Vegetation Index (EVI) correlates with nectar availability^65^. Winter and spring NDVI values have also been shown to influence butterfly population dynamics, such as painted lady butterfly (*Vanessa cardui*) migrations in Africa^67,68^.

### Study time frame and extent

We analyzed LULC data from ~ 2001–2022 as well as climate and NDVI data from 1992–2021 given differences in when these data became available. We provide specific time periods and data used for Mexico and Texas below. We demarcated our study extent in Mexico by intersecting the extent from^4^ with the municipalities in Mexico (Fig. 1). For Texas, we used counties in the US Fish and Wildlife Service (USFWS) Monarch Butterfly Conservation Units^69^ but added coastal counties in Texas (see Ref.^70^ and locations of monarchs observed in the Journey North data (https://journeynorth.org/)). This extent generally agrees with the study extent in Texas used by Ref.^7^ and their modelled kernel densities of monarch larvae. We combined the municipalities in Mexico and counties in Texas for a final study extent (Fig. 1).

### Land cover data

We previously reviewed land cover datasets that could be used in comparisons across Mexico and Texas^64^. For this study, we wanted land cover data with high levels of spatial resolution to track change in habitat that also covered the time series of overwintering abundance in Mexico (1994-present). This requirement necessitated using datasets unique to Mexico and Texas.

For Mexico, we used GLAD data for 2000 and 2020^71^ to track LULC patterns in 8 land cover classes developed from the GLAD land cover strata. GLAD data are currently available for these two years at 30-m resolution. Prior to selecting GLAD, we performed LULC analyses using the European Space Agency Land Cover (ESA-LC) product (1992–2018). This 22-class raster dataset has a 300-m resolution and is produced annually, which seemed ideal for our purposes. However, our analyses indicated the 300-m resolution data did not detect land cover change at acceptable levels of precision (Supplementary results, Fig. S4). We also considered the 30-m resolution North American Land Change Monitoring System (NALCMS) data but at the time we initiated this study, they were only available in 2010 and 2015, though data for 2020 recently became available. Relative to GLAD, both NALCMS and ESA-LC overestimated agriculture in many parts of our Mexico study extent and GLAD maps best-matched aerial imagery (Supplementary results, Fig. S4).

In Texas, we used the 20-class, 30-m resolution NLCD to estimate land cover change from 2001 to 2019 at 7 intervals (2001–2004, 2004–2006, 2006–2008, 2008–2011, 2011–2013, 2013–2016, and 2016–2019) across 13 land cover classes found in our study extent.

Finally, we compared GLAD and NLCD in Texas, where both datasets overlapped, to check how similarly they detected land cover change. For each dataset, we developed maps of percent total gross change in land cover for the bookend years across counties (GLAD, 2000–2020, NLCD, 2001–2019).

### Estimating land cover change

For both Mexico and Texas, we calculated all transitions from one land cover class to another, including no change, by comparing raster data layers in 2001 and 2019 in Texas and 2000 and 2020 in Mexico. For each time interval, we calculated gains, losses, net change, gross change, and annual change for each LULC class. Net change is gain minus loss whereas gross change is gain plus loss. Annual is annualized net change. We summarized land cover change for each municipality (Mexico) or county (Texas) and mapped this change. In addition, we generated chord diagrams of land cover transitions, to visualize the dominant ‘many to one’ changes in land cover classes from the first to last year of the time series.

### Linking land cover change to monarch habitat

Our Monte-Carlo simulations required statistical distributions of milkweed and nectar resources for each land cover class. In Texas, we used the national spring and fall floral resource values from Ref.^72^ to translate land cover to floral resources. Koh et al.^72^ surveyed experts to estimate floral resource values for each season, NLCD class, and crop type in the Cropland Data Layer (CDL) (See SI. pnas.1517685113.sd01.xlsx). NLCD only has a single crop type, so we calculated a weighted average of Koh’s floral values for crops, where weights were the average percent for each CDL crop class making up the NLCD crop class across each of the main NLCD years (2008, 2011, 2013, 2016, Supplementary Data 2). We excluded the grass category due to issues with parsing out sod/seed/grass seed from NLCD grass/hay categories. This arrangement allowed us to use the single cropland class in the NLCD while assigning a nectar value that represented the dominant crops grown across the Texas study extent.

In Mexico, we lacked empirical data of milkweed and nectar abundance among land cover classes. As an alternative, we duplicated the survey developed by Koh et al.^72^ to elicit expert opinion regarding fall milkweed and spring and fall nectar resources in Mexico. The survey (Supplementary results, Figs. S5–S7) was designed and administered by coauthor FB via SurveyMonkey to 30 monarch butterfly experts who participated in the Red Nacional de Monitoreo de la Mariposa Monarca en México National Commission of Protected Area (Working Group for the Conservation and Monitoring of the Monarch Butterfly Flyway). This monitoring program has generated ~ 45,000 monarch sightings, and records monarch behavior on flowering plants, the overnight resting sites on the migratory route, the number of butterflies, and other variables^8^. These biologists were very familiar with INEGI, Mexico’s primary land use and vegetation data^73^, https://www.inegi.org.mx/temas/usosuelo/). The INEGI data contain ~ 135 vegetation and land cover classes, which was too complex for the survey. Instead, we used the land cover classes from a 14-class generalization of the more detailed INEGI vegetation classification system^74^.

The survey asked respondents to provide information about the quality of nectar resources in the spring and the fall, as well as the density of milkweed plants in the fall, for each of the 14 generalized INEGI land cover classes. For nectar, we followed the wording of questions used by Koh et al.^72^, and respondents scored each land cover class with 5 levels of relative resource availability labelled as “0.10”, “0.25”, “0.5”, “0.75”, “0.95”. Even though the 5 levels have quantitative labels, the question used a Likert, or rating scale and the 5 categories represent experts’ subjective opinion about the quality of the nectar resource available for monarchs. The question does not represent a density estimate such as nectar production per unit area. For milkweed, we modified the nectar question slightly, to better address density, because we had density information in Texas. We initially considered asking experts to provide a specific density estimate (plants /ha) for each land cover class to match the units of Texas data, but some experts did not feel comfortable providing this level of detailed information. Instead, we asked respondents to categorize the density of milkweed, as percent cover, across the 5 categories, where each category label represented a density class (0.10 = 10%, Fig. S8, Supplementary Results). Given this limitation, the milkweed density data in Mexico provide relative differences across land cover classes, but do not allow us to measure plants per ha, or convert density to an estimate of total plants, as we did in Texas.

The data were normally distributed so means and standard deviations were calculated across respondents for each land cover class. Anonymized survey results are available in Supplementary Data 3.

Different land cover datasets are rarely fully in agreement. To incorporate the uncertainty between the 14-class INEGI used in the survey and the 8-class GLAD used for land cover change, we used agreement matrices between INEGI and GLAD to translate the survey-based scores for milkweed and floral resources, based on INEGI classes, to the GLAD classes (for an example with the GLAD forest class, see Supplementary results, Table S1). Each GLAD class comprised different proportions of the INEGI land cover classes. We used these proportions to calculate a weighted average of both the mean and standard deviation of the survey-based scores for each GLAD class. We averaged the agreement/disagreement proportions between GLAD and INEGI versions (series 3, 4, 5 and 6) and each year of GLAD, and used these values when calculating the weighed means and standard deviations of the survey-based nectar and milkweed scores (Supplementary Data 4).

For spring milkweed in Texas, we analyzed milkweed density (plants/ha) data (n = 613) from a variety of sources compiled by the Xerces Society for U.S. Geological Survey (these data are in Supplementary Data 5), 33 of the 613 samples were proprietary IMMP data collected in Texas (provided by the Monarch Joint Venture and USFWS). Xerces contacted 140 state biologists and university researchers to generate the data. These data represent different field efforts designed to sample milkweed density. They were collected in 2004–7, 2009, 2011, 2015, and 2016. Data were filtered to include samples that were in Texas, had geographic coordinates and were density estimates (plants/ha) not presence/absence data. In addition, we excluded data with comments that called their quality into question. For example, some excluded records noted “student maxed out at 15” indicating a plot had more than 15 plants, but the field technician stopped counting at 15. The remaining 613 samples were overlaid with NLCD, then manually checked with aerial imagery. The NLCD landcover classes were adjusted when aerial imagery indicated a misclassification of the land cover. This adjustment happened mainly along roads, where natural lands were misclassified as developed.

Across the entire state, and within each land cover class, the milkweed density data were highly skewed, dominated by zeroes (60–91% across land cover classes). We selected the best fitting distribution for each land cover class, by comparing zero-inflated binomial, exponential, negative binomial, and zero-inflated Poisson distributions (Supplementary Data 7). Despite > 600 samples, six land cover classes had low sample sizes for estimating milkweed densities. Five of the classes, Open Water, Barren Land, Cultivated Crops, Woody Wetlands and Herbaceous Wetlands, had no milkweeds present in the samples. Lacking better data, we set these values to zero. The final land cover class with sparse data, Developed Medium Intensity, was estimated as the midpoint between the Low- and High-Density Developed classes.

Note that the survey-based estimate of floral resources (spring and fall in Texas and Mexico) and milkweed (spring, Mexico) are unitless, representing expert opinion of suitability on a scale from 0 to 1. Adding these across pixels creates total values that are difficult to interpret, so we report these data as percent change, and they represent relative differences through time. In Texas, the spring milkweed data represent actual plant density (plants/ha), and we report changes in this value, as total plants, as well as percent change.

### Monarch habitat change

To estimate extent-wide changes in monarch habitat in Mexico from 2000 to 2020 and Texas from 2001 to 2019, we combined the area estimates of change in each land cover class with the distributions of nectar value or milkweed abundance for each land cover class using Monte-Carlo simulation. For milkweed density in Texas, we used the best fitting distribution among those we evaluated. For Texas nectar, Koh et al.^72^ reported parameter values from continuous beta probability distributions, which we used. In Mexico, as noted above, survey data were normally distributed and we used means and standard deviations for each LC class to generate distributions for spring milkweed and spring/fall nectar.

For 2000 and 2020 in Mexico, and 2001 and 2019 in Texas, we first multiplied the areal amount of change by a random draw from the distribution of floral resource or milkweed value for the respective season and land cover class. We then summed the nectar or milkweed value across all land cover classes to estimate an extent-wide value for each study year and then subtracted the first year from the last year to estimate change from 2000 to 2020 in Mexico and from 2001 to 2019 in Texas. This process was repeated 5,000 times and we report the mean and standard error of the change and the percent change from the 5,000 draws from the start and end years in Mexico and Texas.

To visualize spatial patterns of change across the entire study extent, and identify areas of increasing or decreasing habitat, we mapped the amount of change in nectar and milkweed resource value across municipalities (Mexico) and counties (Texas). Because the milkweed resource values data were on different scales (field-based measurements in Texas vs expert opinion in Mexico), we fit the estimated mean change of milkweed density in Mexico to the range established in the Texas field data. To do so, we calculated a z-score of the Mexico milkweed density change values, their means, and their standard deviations, and then subsequently de-standardized those values by using the mean and standard deviation of the Texas milkweed density change values. The result is a range of milkweed change values across Texas and Mexico all within the same range of values.

### Changes in climate and NDVI

To estimate climate trends in the spring and fall, we gathered data from Google Earth Engine (GEE), summarized the data into seasonal median values for each year, and used linear models to estimate trends through time. We developed Python^75^ scripts to extract TerraClimate data and Landsat derived NDVI from 1992 to 2021, with the GEE API and geemap Python library (https://geemap.org). All data were sourced from the GEE database (https://developers.google.com/earth-engine/datasets). Data from TerraClimate (https://www.climatologylab.org/terraclimate.html) included precipitation (precip, mm), maximum temperature (tmax, °C + 10), minimum temperature (tmin, °C + 10), and Palmer Drought Severity Index (pdsi, unitless), at a resolution of 4638.3 m. NDVI data came from (https://www.usgs.gov/centers/eros/science/usgs-eros-archive-vegetation-monitoring-noaa-cdr-ndvi), and provide a measure of vegetation greenness, an important indicator of vegetation health. NDVI data have a 30 m resolution but were summarized at a resolution of 300 m.

We split the climate and NDVI data into fall (September, October, and November) and spring (March, April, and May) and analyzed these separately. For each year, we calculated a median across all available estimates within a season using the median reducer from GEE. For example, satellites collect NDVI every 8 days, so for a 3-month season (~ 90 days), we had ~ 11, 8-day values from which we calculated a median. This was done for each pixel, for each year, to create a median composite NDVI image across the entire study extent. We repeated this process, for each variable, for every year from 1992 to 2021.

For each year, we summarized the map across the study extents for Mexico and Texas by calculating a median across all pixels. The median value was then assembled into a time series, forming a set of climate and NDVI data that covered ~ 30-year period in Texas and Mexico. We used simple linear regressions to check for temporal trends where the climate variable or NDVI was the dependent variable and year the explanatory variable.

In addition to the supplemental data included in the paper, data and code associated with the analyses are available at Ref.^76^.

### Supplementary Information

Supplementary Information 1.

Supplementary Information 2.

Supplementary Information 3.

Supplementary Information 4.

Supplementary Information 5.

Supplementary Information 6.

Supplementary Information 7.

## Data Availability

The datasets used and/or analyzed during the current study available from the corresponding author on reasonable request. The datasets generated and/or analyzed during the current study are available in the USGS ScienceBase repository at 10.5066/P1MUA57V.
