# Supplementary material for: Changes in landscape and climate in Mexico and Texas reveal small effects on migratory habitat of monarch butterflies (Danaus plexippus)
Source: Sci Rep. 2024 Mar 20;14:6703. doi: 10.1038/s41598-024-56693-z (PMC10954652; doi:10.1038/s41598-024-56693-z)
Supplement: Supplementary file 7 — Supplementary Information 7. [file 41598_2024_56693_MOESM7_ESM.docx]

**Fig. S1.**

Total percent land cover change, measured as the percent of pixels within a county that showed any change for the National Land Cover Database (NLCD, 2001 - 2019) (upper panel) and Global Land Analysis & Discovery (GLAD, 2000 - 2020) (lower panel) created using ArcGIS Pro 3.2.1, Copyright © 1995-2023 ESRI (<https://www.esri.com/en-us/arcgis/products/arcgis-pro/overview>).


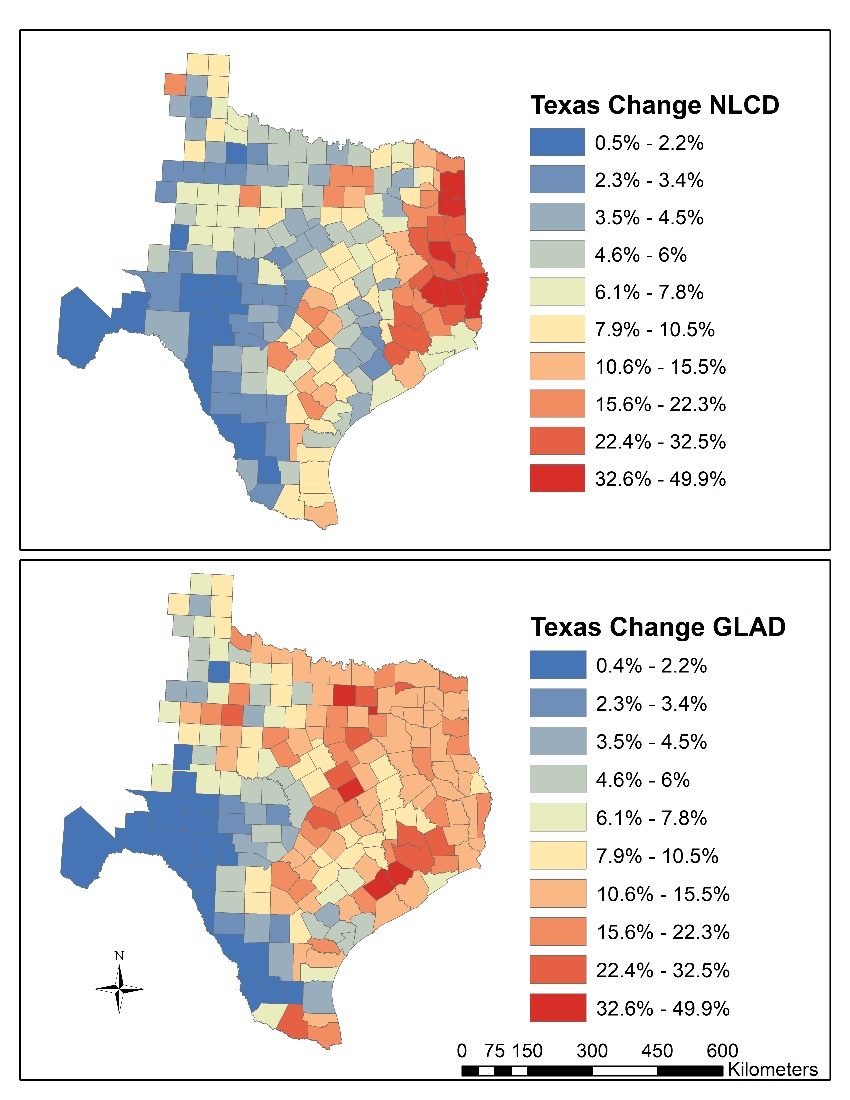


**Fig. S2.** **Mexico climate variables and NDVI.** Time series of environmental variables for the Fall or Spring across the Mexico study extent from 1992 to 2021. Normalized Difference Vegetation Index (NDVI) was analyzed from 1997 to 2021. Each point represents that year’s seasonal median value.
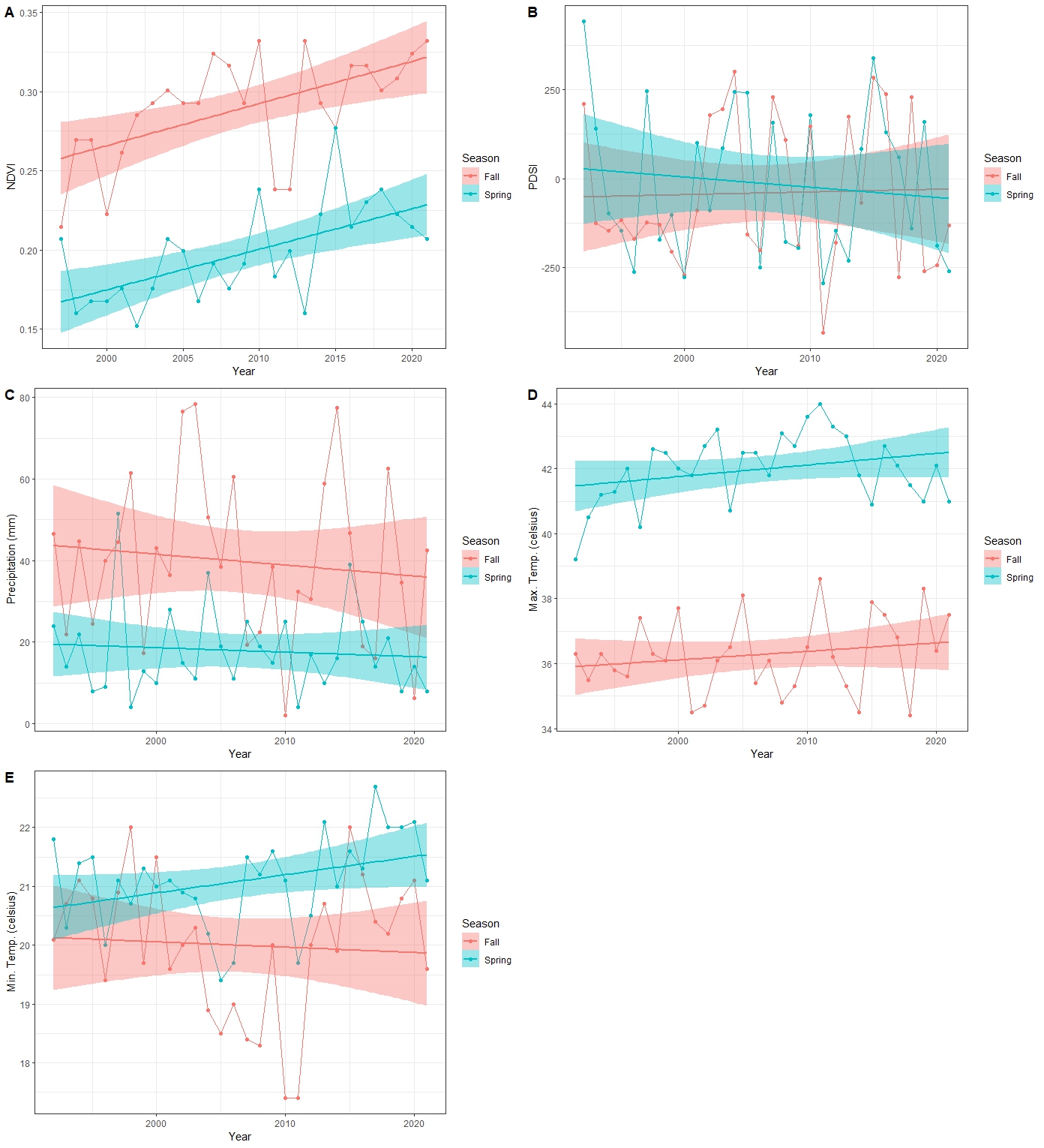


**Fig. S3. Texas climate variables and NDVI.** Time series of environmental variables for the Fall or Spring across the Mexico study extent from 1992 to 2021. Normalized Difference Vegetation Index (NDVI) was analyzed from 1997 to 2021. Each point represents that year’s seasonal median value.


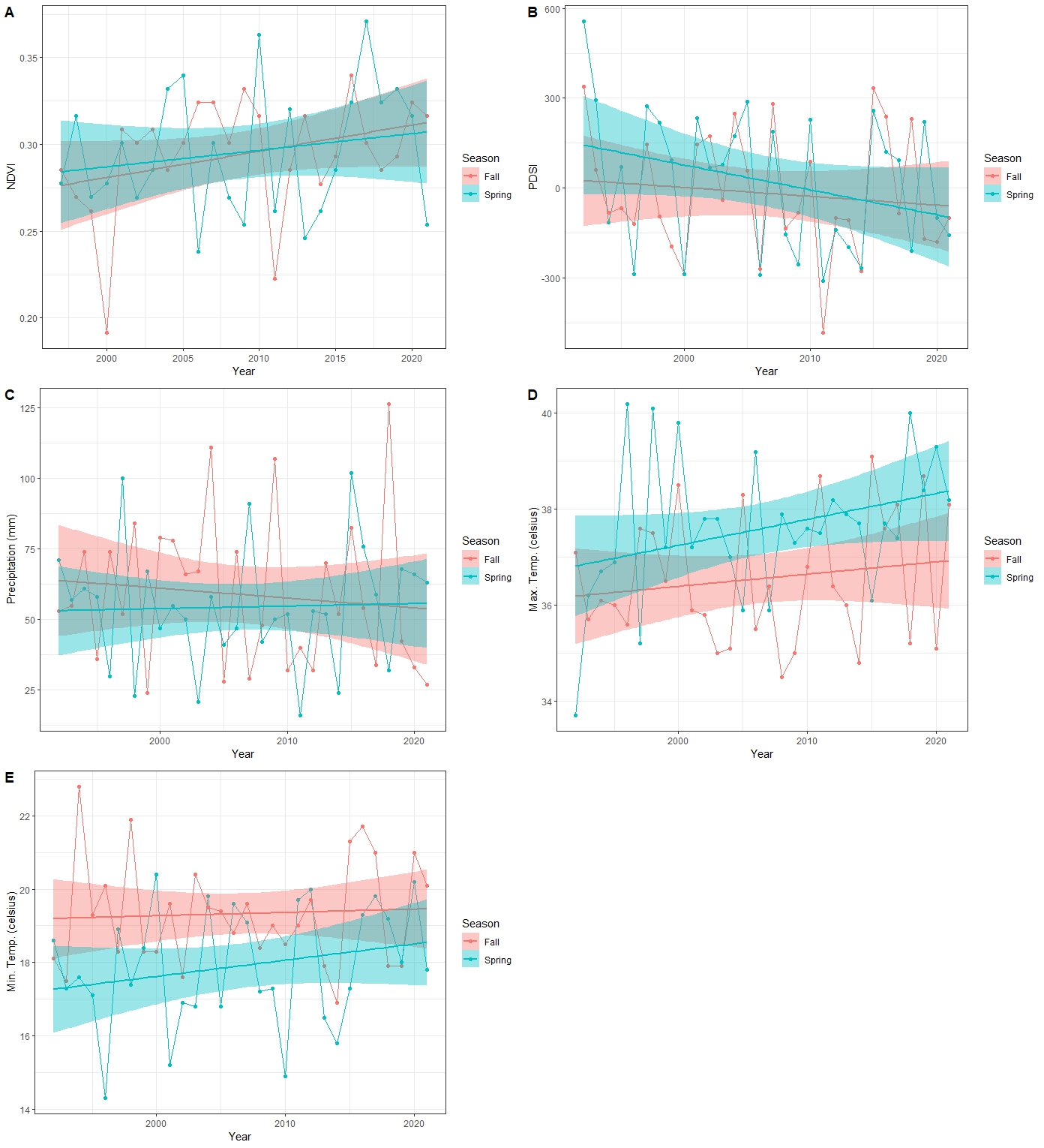


**Fig. S4.**

Aerial Imagery (ESRI basemap World Imagery [https://services.arcgisonline.com/ArcGIS/rest/services/World_Imagery/MapServer](https://gcc02.safelinks.protection.outlook.com/?url=https%3A%2F%2Fservices.arcgisonline.com%2FArcGIS%2Frest%2Fservices%2FWorld_Imagery%2FMapServer&data=05%7C02%7Cjediffendorfer%40usgs.gov%7C541c76df31384e09754d08dc3df6a0a4%7C0693b5ba4b184d7b9341f32f400a5494%7C0%7C0%7C638453376669419763%7CUnknown%7CTWFpbGZsb3d8eyJWIjoiMC4wLjAwMDAiLCJQIjoiV2luMzIiLCJBTiI6Ik1haWwiLCJXVCI6Mn0%3D%7C0%7C%7C%7C&sdata=acvKVf6AbofJDqSMcEfs6A%2Bds61deEacTj4PBnI%2BAb0%3D&reserved=0)) (A) and maps of land use and cover from Global Land Analysis & Discovery (GLAD) ([https://glad.umd.edu/dataset/GLCLUC2020](https://gcc02.safelinks.protection.outlook.com/?url=https%3A%2F%2Fglad.umd.edu%2Fdataset%2FGLCLUC2020&data=05%7C02%7Cjediffendorfer%40usgs.gov%7C541c76df31384e09754d08dc3df6a0a4%7C0693b5ba4b184d7b9341f32f400a5494%7C0%7C0%7C638453376669429377%7CUnknown%7CTWFpbGZsb3d8eyJWIjoiMC4wLjAwMDAiLCJQIjoiV2luMzIiLCJBTiI6Ik1haWwiLCJXVCI6Mn0%3D%7C0%7C%7C%7C&sdata=d0%2BrtpQ%2FAD49Y%2BuGzguCudig9jQ%2FTd%2B0bDMBDT7GVEc%3D&reserved=0)) (B), European Space Agency-Land Cover (ESA-LC) ([https://maps.elie.ucl.ac.be/CCI/viewer/download.php](https://gcc02.safelinks.protection.outlook.com/?url=https%3A%2F%2Fmaps.elie.ucl.ac.be%2FCCI%2Fviewer%2Fdownload.php&data=05%7C02%7Cjediffendorfer%40usgs.gov%7C541c76df31384e09754d08dc3df6a0a4%7C0693b5ba4b184d7b9341f32f400a5494%7C0%7C0%7C638453376669436730%7CUnknown%7CTWFpbGZsb3d8eyJWIjoiMC4wLjAwMDAiLCJQIjoiV2luMzIiLCJBTiI6Ik1haWwiLCJXVCI6Mn0%3D%7C0%7C%7C%7C&sdata=K4F31KZ13sy4RxyOXbBpYkmEREEkSUkTHC3KsSBXPD8%3D&reserved=0)) (C), and North American Land Change Monitoring System (NALCMS) ([https://www.mrlc.gov/data?f%5B0%5D=category%3Aland%20cover](https://gcc02.safelinks.protection.outlook.com/?url=https%3A%2F%2Fwww.mrlc.gov%2Fdata%3Ff%255B0%255D%3Dcategory%253Aland%2520cover&data=05%7C02%7Cjediffendorfer%40usgs.gov%7C541c76df31384e09754d08dc3df6a0a4%7C0693b5ba4b184d7b9341f32f400a5494%7C0%7C0%7C638453376669442663%7CUnknown%7CTWFpbGZsb3d8eyJWIjoiMC4wLjAwMDAiLCJQIjoiV2luMzIiLCJBTiI6Ik1haWwiLCJXVCI6Mn0%3D%7C0%7C%7C%7C&sdata=JtnFt5cSy%2FUYaZQ7u8DXUaJIasf38zFfmegNT%2Faxu00%3D&reserved=0)) (D) for the same region of Mexico. GLAD performed best, capturing detailed heterogeneity in shrub/grass and croplands that was largely missed by the lower resolution ESA-LC. GLAD also performed better than NALCMS (D) for major terrestrial LC classes (grass/shrub/forest/croplands). This map was created using ArcGIS Pro 3.2.1, Copyright © 1995-2023 ESRI (<https://www.esri.com/en-us/arcgis/products/arcgis-pro/overview>).


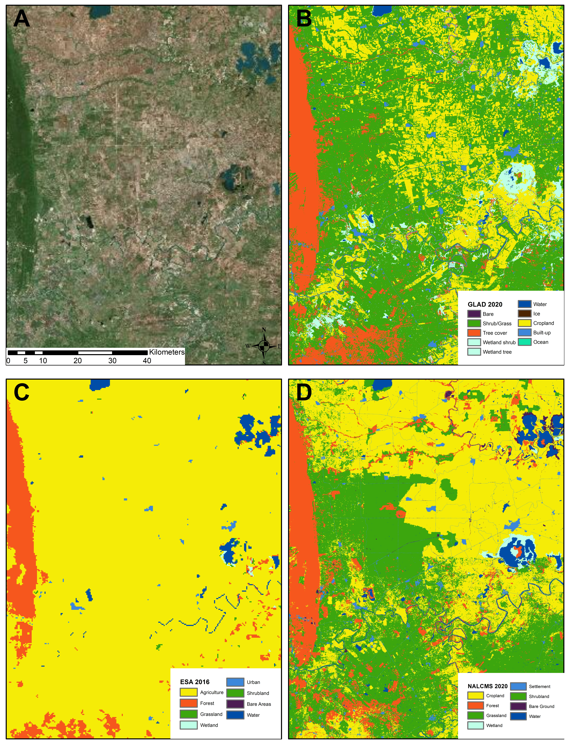


**Fig. S5.**

Figure S5 translation. 1.What is your name? 2. Where do you habitually perform monarch monitoring (Please include the following information: location, municipality, state. If you do it in different places you can separate with semicolons, for example: San Miguel, Tepezala, Aguascalentes; San Juan de los Lagos, San Juan de los Lagos, Jalisco). 3. In what months have you seen and made reports of monarch butterflies (For example, October and November). 4. How many years have you been making reports of monarch butterflies? 5. Approximately how many reports have you made on monarch butterflies?


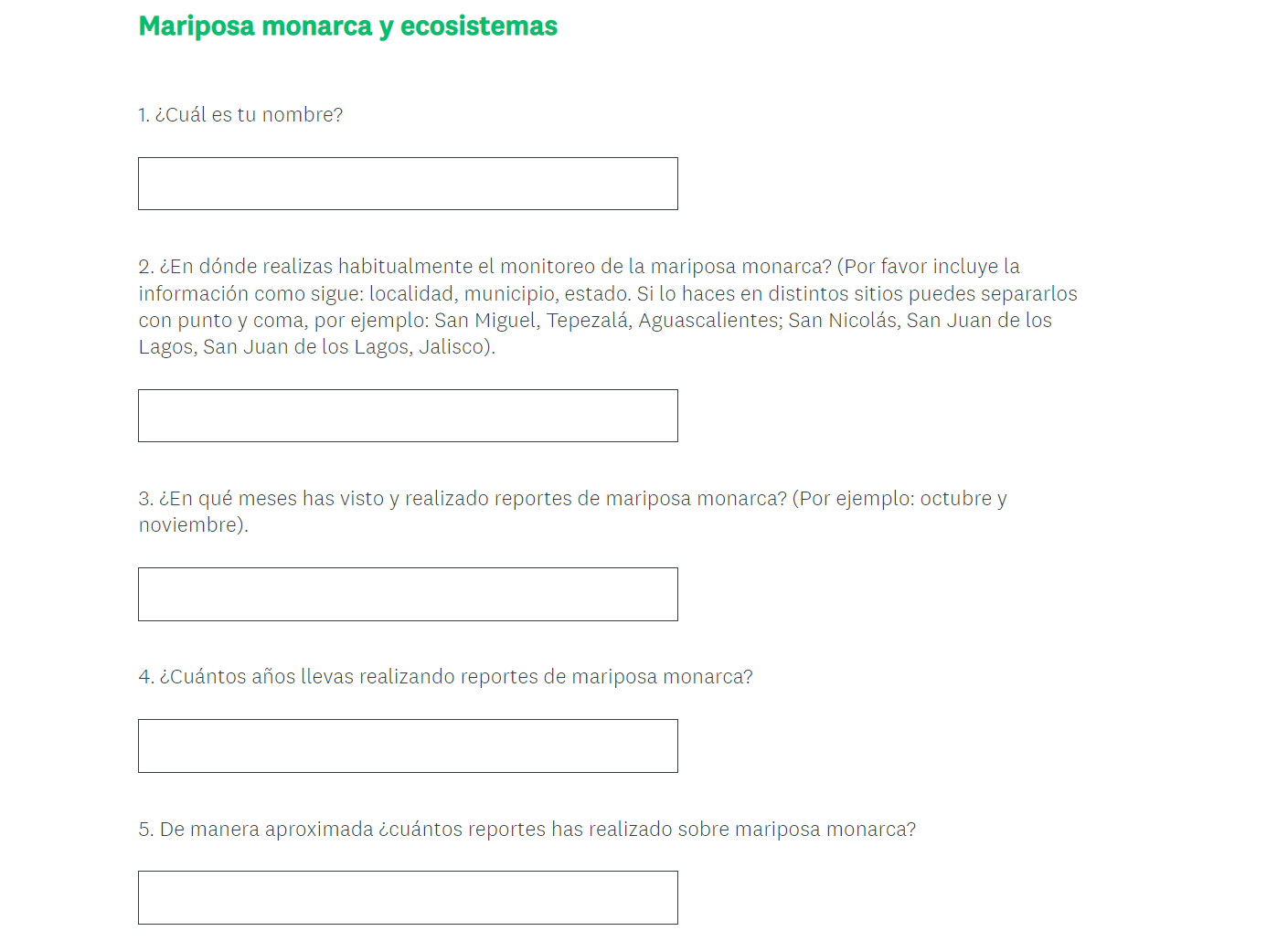


**Fig. S6.**

Figure S6 translation. 6. For the Fall season, please rate the quality of floral resources that butterflies could find in each of the following ecosystems according to the following categories 0.10; 0.25; 0.50; 0.75; 0.95 (0.1 being the least relative amount of nectar available when butterflies pass through said ecosystem and 0.95 being the greatest relative amount of nectar available).


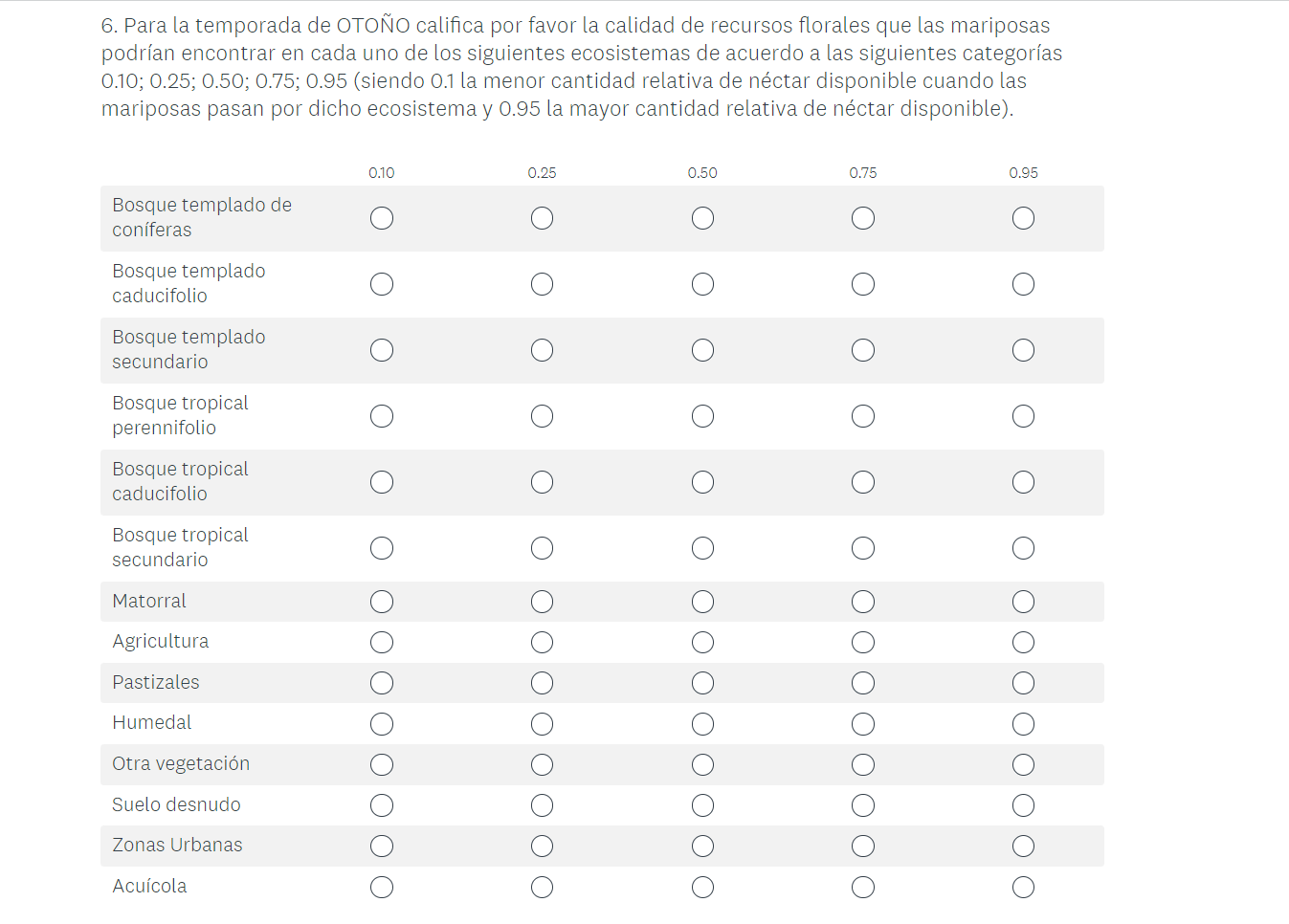


**Fig. S7.**

Figure S7 translation. 7. For the spring season, please rate the quality of floral resources that butterflies could find in each of the following ecosystems according to the following categories 0.10; 0.25; 0.50; 0.75; 0.95 (0.1 being the least relative amount of nectar available when butterflies pass through said ecosystem and 0.95 being the greatest relative amount of nectar available)


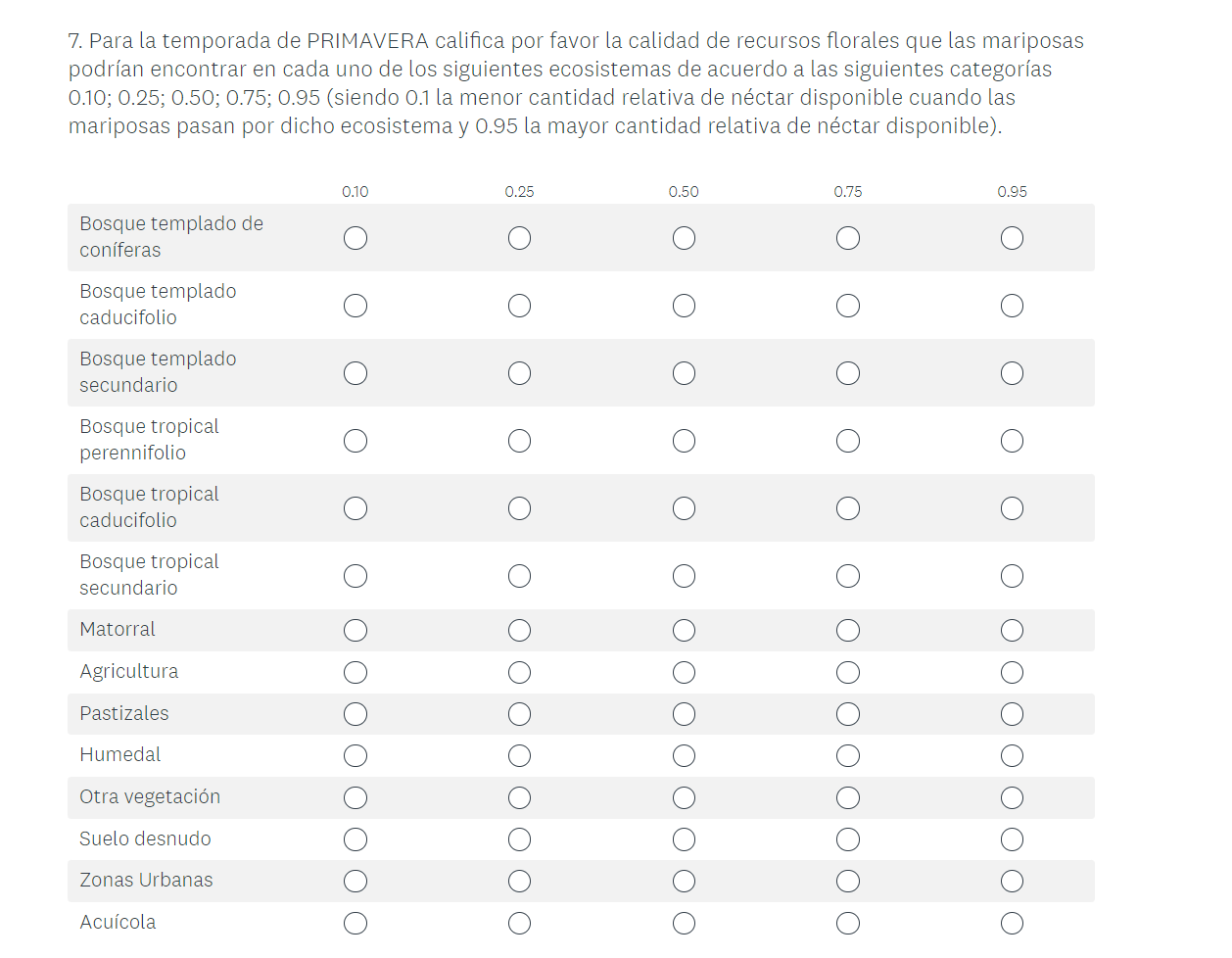


**Fig. S8.**

Figure S8 translation. 8. For the Spring season, in each of the following ecosystems what is the density of stems of Asclepias spp. that you encounter, on average, for a typical hectare of vegetation” Categories 0.10; 0.25; 0.50; 0.75; 0.95 (0.1 corresponds to 10% of the area is covered by Asclepias spp, and consecutively). 9. Thank you very much for your participation. Do you have any comments?


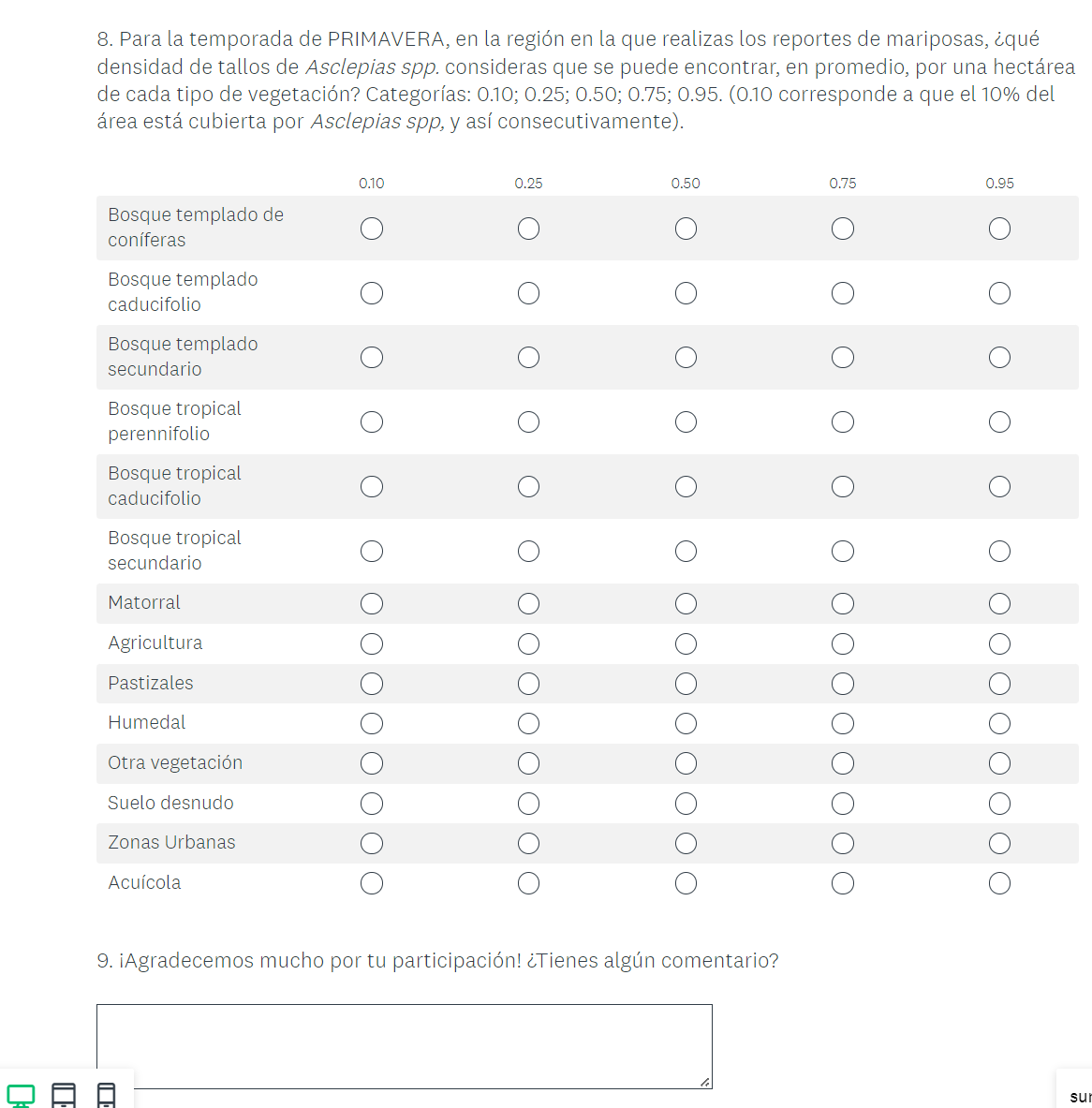


**Table S1.**

Example of the workflow for adjusting the survey mean scores for each INEGI class to GLAD classes for the forest class in GLAD. Survey mean is the mean of the unadjusted scores across respondents for spring Nectar. 2000 and 2020 Crosswalk proportion, show the proportion of each INEGI landcover class occurring in the GLAD forest class across the entire MX study extent, thus offering a measure of congruence between the two classification systems. The Survey mean is multiplied by Crosswalk proportion to generate a 2000 and 2020 weighted score. These two years are then averaged to an Average weighted score. The sum of Average weighted score across all INEGI land cover classes that occur into the GLAD forest class is the adjusted spring nectar value for GLAD (0.404). This process was applied to all the GLAD land cover classes for spring and fall Nectar.

| INEGI Class | Survey mean | 2000 Crosswalk proportion | 2020 Crosswalk proportion | 2000 weighted score | 2020 weighted score | Average weighted score |
| --- | --- | --- | --- | --- | --- | --- |
| Coniferous Temperate Forest | 0.36 | 0.126630 | 0.127250 | 0.045587 | 0.045810 | 0.046000 |
| Deciduous Temperate Forest | 0.37 | 0.235627 | 0.236680 | 0.087182 | 0.087571 | 0.087000 |
| Deciduous Tropical Forest | 0.52 | 0.070182 | 0.065392 | 0.036495 | 0.034004 | 0.035249 |
| Evergreen Tropical Forest | 0.46 | 0.015061 | 0.015139 | 0.006928 | 0.006964 | 0.006946 |
| Secondary Temperate Forest | 0.41 | 0.144670 | 0.160334 | 0.059315 | 0.065737 | 0.063000 |
| Secondary Tropical Forest | 0.41 | 0.114852 | 0.127562 | 0.047089 | 0.052301 | 0.049695 |
| Agriculture | 0.45 | 0.137178 | 0.124413 | 0.061730 | 0.055986 | 0.058858 |
| Bare Ground | 0.16 | 0.000066 | 0.000119 | 0.000011 | 0.000019 | 0.000015 |
| Urban/Human | 0.37 | 0.000597 | 0.001050 | 0.000221 | 0.000388 | 0.000305 |
| Other/Sparse Vegetation | 0.32 | 0.000232 | 0.000108 | 0.000074 | 0.000034 | 0.000054 |
| Scrub/Shrub | 0.44 | 0.066578 | 0.065180 | 0.029294 | 0.028679 | 0.028987 |
| Wetland | 0.37 | 0.000191 | 0.000193 | 0.000071 | 0.000071 | 0.000071 |
| Grassland | 0.34 | 0.087694 | 0.076144 | 0.029816 | 0.025889 | 0.027852 |
| Totals | N/A | 1 | 1 | N/A | N/A | 0.404 |
